# Prediction of dementia risk by instrumental activities of daily living limitations and its impact on dementia onset in combination with mild cognitive impairment: a population-based longitudinal study

**DOI:** 10.1186/s12889-025-22788-z

**Published:** 2025-04-25

**Authors:** Keitaro Makino, Sangyoon Lee, Osamu Katayama, Kouki Tomida, Ryo Yamaguchi, Daiki Yamagiwa, Hiroyuki Shimada

**Affiliations:** 1 Center for Environmental and Health Sciences, Hokkaido University, North-12, West-7, Kita-ku, Sapporo, 060-0812 Japan; 2 Department of Preventive Gerontology, Center for Gerontology and Social Science, National Center for Geriatrics and Gerontology, Obu, Japan

**Keywords:** Instrumental activities of daily living, Mild cognitive impairment, Dementia, Older adults

## Abstract

**Background:**

Instrumental activities of daily living (IADL) limitations are important risk factors for dementia. IADL is lifestyle-based, therefore, its assessment content must be updated to reflect recent lifestyle changes. We evaluated the predictive validity of the National Center for Geriatrics and Gerontology Activities of Daily Living (NCGG-ADL), an IADL scale we previously developed, to identify dementia risk and the combined impact of mild cognitive impairment (MCI) and IADL limitations on dementia onset.

**Methods:**

This population-based longitudinal study included 2,118 adults aged ≥ 65 years without dementia at baseline. At baseline, IADL limitations and MCI were assessed using the NCGG-ADL and a multi-domain neuropsychological test, respectively. The participants were followed up for new-onset dementia monthly for 5 years.

**Results:**

Among all participants, 247 (11.7%) had IADL limitations at baseline, and 151 (7.1%) developed dementia during follow-up. Compared to participants without IADL limitations (NCGG-ADL 13 points), those with IADL limitations (NCGG-ADL ≤ 12 points) showed a significantly higher dementia onset risk (HR: 1.55 [95% CI: 1.04–2.31]). Regarding the combined impact of MCI and IADL limitations on dementia, the HRs (95% CIs) (reference: unimpaired cognition without IADL limitations) of unimpaired cognition with IADL limitations, MCI without IADL limitations, and MCI with IADL limitations were 1.53 (0.90–2.61), 1.87 (1.28–2.74), and 2.88 (1.65–5.03), respectively.

**Conclusions:**

This study validated the NCGG-ADL as an effective screening tool for assessing dementia risk among community-dwelling older adults. Furthermore, concomitant MCI and IADL limitations was associated with a higher risk of dementia onset more than either condition alone. Therefore, this coexisting condition should be carefully monitored to prevent dementia.

**Supplementary Information:**

The online version contains supplementary material available at 10.1186/s12889-025-22788-z.

## Background

The prevalence of dementia is rapidly increasing with the aging population. According to predictions, the total number of people with dementia will reach 82 million by 2030 and 152 million by 2050 [1], posing a heavy social and economic burden. Therefore, the risk factors for dementia must be identified and addressed earlier to prevent dementia.

Early prevention of dementia can be achieved during the occurrence of mild cognitive impairment (MCI), which represents a transitional phase between normal cognitive aging and dementia [2]. The commonly used criteria of MCI include concerns over changes in cognition, impairment in one or more cognitive domains, and preservation of independence in functional abilities [3]. However, individuals with MCI experience a certain degree of functional impairment [3, 4], particularly in instrumental activities of daily living (IADLs) [5, 6].

In general, functional capacity in older adults is divided into basic activities of daily living (BADLs), which include self-maintenance skills such as dressing, eating, and bathing, and IADLs, which include more complex and higher functional abilities, such as shopping, managing money, and using public transportation [5, 7]. IADLs demand more complex cognitive functioning than BADLs [4] and are more susceptible to subtle deterioration associated with cognitive decline [8]. In addition, MCI combined with IADL limitations is associated with a higher probability of dementia onset and a lower chance of recovery to normal cognition [3, 9]. Therefore, the assessment of IADLs in individuals without dementia is considered useful in predicting dementia risk and provides important information to support timely intervention [10].

There are various reliable and valid scales for assessing IADLs [7, 11, 12]. However, demographic and economic changes have affected family structures and individual lifestyles in recent decades. For example, the use of mobile phones, computers, and household appliances has become a part of everyday life [13]. However, operational capabilities regarding electrical appliances have not been sufficiently assessed in the above-mentioned scales. A recent systematic review examining the relationship between IADLs and cognitive function highlighted the need to include technology-related items in IADL assessments [5]. Therefore, we previously developed an IADL scale named the National Center for Geriatrics and Gerontology Activities of Daily Living (NCGG-ADL) [14], which comprised a 13-item self-report questionnaire regarding individuals’ abilities to conduct IADL tasks that allows for the discrimination of a wide range of IADL levels that correspond to a recent lifestyle, including the operation of electrical appliances [14]. The NCGG-ADL is simple and highly reliable (Cronbach’s α = 0.937) and has a cut-off point (12/13 points) to predict new incidences of functional disability [14]. However, the predictive validity of the NCGG-ADL for detecting new-onset dementia has not yet been examined.

We aimed to confirm the predictive validity of IADL abilities assessed using the NCGG-ADL in detecting dementia risk and examined the combined impact of MCI and IADL limitations on dementia onset.

## Methods

### Study setting and participants

This longitudinal cohort study included community-dwelling older adults enrolled in a sub-cohort of the National Center for Geriatric and Gerontology–Study of Geriatric Syndromes (NCGG-SGS). The inclusion criteria were residence in Takahama City, Aichi Prefecture, Japan, and age ≥ 60 years at baseline. A total of 4,167 individuals participated in a baseline on-site survey (September 2015 to February 2017), which included data collection on IADLs. After the baseline survey, we excluded participants with the following characteristics: (1) prior diagnosis of dementia at baseline and/or severe cognitive impairment based on Mini-Mental State Examination (MMSE) [15] scores < 24 (*n* = 390) [16]; (2) history of stroke and/or depression and/or other brain diseases (*n* = 377); (3) missing data for the above criteria and assessment of cognitive performance and/or IADLs (*n* = 86); and (4) participants aged < 65 years (*n* = 700) as our follow-up of dementia incidence used data of the National Long-Term Care Insurance System that applies to Japanese adults aged ≥ 65 years. After exclusion, 2,614 individuals without dementia at baseline were considered potential participants for the monthly follow-up of dementia onset. During the follow-up period, we excluded participants who did not have available data on the Long-Term Care Insurance System and/or National Health Insurance Systems for over 5 years (*n* = 496), and 2,118 individuals were included in our longitudinal analysis.

The study was conducted in accordance with the principles of the Declaration of Helsinki. The Ethics Committee of the National Center for Geriatrics and Gerontology approved the study protocol (Approval Number: 1440–5). Informed consent was obtained from all participants before their inclusion in the study.

### Assessment of IADL

The self-reported IADL ability was assessed at baseline using the NCGG-ADL [14]. The NCGG-ADL contains questions on 13 daily activities: (1) cutting toenails; (2) going out by oneself; (3) taking a bus or train; (4) shopping for necessities; (5) transferring money; (6) looking up a telephone number; (7) vacuuming; (8) managing money; (9) controlling medications; (10) managing a house key; 11) cooking; 12) using a microwave; and 13) using a gas stove. Participants reported their abilities to independently conduct each of the 13 activities over the past month using a simple dichotomous rating (yes/no). The score was calculated by summing the number of “Yes” responses (0–13), with higher scores indicating higher IADL abilities [14]. Participants were divided into those with IADL limitations (NCGG-ADL ≤ 12 points) and those without IADL limitations (NCGG-ADL 13 points) according to previously established cut-off points [14].

### Assessment of MCI

Cognitive function was assessed using a tablet-based multi-domain neuropsychological test, the National Center for Geriatrics and Gerontology-Functional Assessment Tool (NCGG-FAT) [17]. The NCGG-FAT comprises the following cognitive domain tests: (1) memory (word list memory-I [immediate recognition] and word list memory-II [delayed recall]); (2) attention (Trail Making Test-part A); (3) executive function (Trail Making Test-part B); and (4) processing speed (Digit Symbol Substitution Test). The NCGG-FAT has high test-retest reliability and moderate-to-high validity in community-dwelling older adults [17]. All tests have established standardized thresholds for defining the objective cognitive impairments in their respective domains; the threshold used was a score of ≥ 1.5 standard deviations below the age- and education-specific mean, which was derived from a population-based database of over 10,000 older adults [18].

We defined MCI using the following criteria: (1) absence of dementia, (2) functional independence in BADL (eating, bathing, grooming, walking, and stair-climbing), (3) maintenance of global cognitive functioning (MMSE ≥ 24 points), and (4) objective cognitive impairment (below the reference threshold described above in one or more cognitive domains including memory, attention, executive function, and processing speed) [19, 20]. Based on the above criteria, participants were classified as having unimpaired cognition or MCI [20].

### Follow-up of dementia onset

We followed the participants for 5 years for dementia onset using data records of the Public Health Insurance and the Long-Term Care Insurance.

Regarding the Public Health Insurance systems in Japan, all adults aged ≥ 65 years have either of the following health insurance: health insurance for employed individuals (Employees’ Health Insurance), national health insurance for unemployed and self-employed individuals aged < 75 years (Japanese National Health Insurance), or health care for individuals aged ≥ 75 years (Later-Stage Medical Care System) [21]. We tracked the records of the Japanese National Health Insurance and Late-Stage Medical Care System for new dementia cases [16]. Dementia was diagnosed by a physician according to the International Classification of Disease-10 (ICD-10) codes [16]. The details of our criteria for categorizing ICD-10 codes using NCGG-SGS data have been described elsewhere [22].

The Long-Term Care Insurance is a mandatory form of social insurance that supports the daily lives of older adults with functional disabilities in Japan [23]. All individuals aged ≥ 65 years are eligible for institutional or community-based services, depending on their disability level. The disability certification process comprises (i) the degree of disability based on a questionnaire developed by the Ministry of Health, Labour and Welfare of Japan and (ii) a physician’s written opinion prepared by the attending physician [24]. We identified dementia using the records of Dementia Rating Scale scores (level of independence in daily living for older adults with dementia) in the above disability certification process [25, 26]. The details of our criteria for categorizing the Dementia Rating Scale score using NCGG-SGS data have been described previously [22].

We reviewed participants monthly for new-onset dementia for 5 years and defined dementia onset as a new record of dementia in either the Public Health Insurance database or the Long-Term Care Insurance database [22].

### Potential confounding factors

We assessed demographic and health-related characteristics including age, sex, educational level, and medical history (hypertension, diabetes mellitus, and heart disease) as potential confounding factors. Trained nurses assessed the medical histories based on self-reports through face-to-face interviews. We also included global cognitive function assessed using the MMSE [15] at baseline as a covariate.

### Statistical analyses

We compared the baseline characteristics between participants with and without IADL limitations and between those with and without dementia onset using Student’s t-test for continuous variables and the chi-squared test for categorical variables. Further, we divided the participants into four groups according to the combination of MCI and IADL limitations: unimpaired cognition without IADL limitations, unimpaired cognition with IADL limitations, MCI without IADL limitations, and MCI with IADL limitations. The cumulative survival rates for dementia onset during the 5-year follow-up period in each group were calculated using Kaplan–Meier curves. Intergroup differences were estimated using the log-rank test, and multiple comparisons were corrected using the Shaffer procedure. Moreover, Cox proportional hazards regression analysis was conducted to examine the effects of IADL limitations and its combined impact with MCI on dementia onset. The proportional hazards assumption was verified by checking a log-log plot, and no violations were detected. Hazard ratios (HRs) for the risk of dementia onset were calculated with 95% confidence intervals (CIs). As a sensitivity analysis, we conducted Cox regression analyses on IADL ability and dementia risk only in participants with MMSE scores ≥ 28, indicating intact cognition [27], to address the possibility of misclassification in self-reported IADL ability.

The level of statistical significance was set at a two-sided P-value of < 0.05. All statistical analyses were performed using IBM SPSS Statistics version 25 (IBM Japan, Tokyo, Japan).

## Results

### Participant characteristics

Of the 2,118 enrolled participants, 247 (11.7%) had IADL limitations based on the NCGG-ADL at baseline, and 151 (7.1%) developed dementia within 5 years.

Table 1 provides the differences in characteristics between the participants with and without IADL limitations. At baseline, participants with IADL limitations were significantly older (*P* < 0.001), more likely to be men (*P* < 0.001), had lower education levels (*P* < 0.001), had lower global cognitive functions (*P* < 0.001), and had a higher prevalence of MCI (*P* < 0.001) than participants without IADL limitations. Moreover, the incidence of dementia during the 5 years was significantly higher in participants with IADL limitations than in those without (*P* < 0.001).

**Table 1:** Participant characteristics according to instrumental activities of daily living ability at baseline

Variables	Overall *n* = 2,118	IADL ability at baseline		*P*-value ^*^
		Participants without IADL limitations (NCGG-ADL 13 points) *n* = 1,871	Participants with IADL limitations (NCGG-ADL ≤ 12 points) *n* = 247	
Age, years	73.5 ± 5.9	73.1 ± 5.7	76.2 ± 6.4	< 0.001
Sex, men (%)	841 (39.7)	686 (36.7)	155 (62.8)	< 0.001
Education, years	11.0 ± 2.3	11.1 ± 2.3	10.4 ± 2.2	< 0.001
Hypertension, n (%)	1,067 (50.4)	935 (50.0)	132 (53.4)	0.306
Diabetes mellitus, n (%)	302 (14.3)	257 (13.7)	45 (18.2)	0.058
Heart disease, n (%)	348 (16.4)	298 (15.9)	50 (20.2)	0.085
MMSE, points	27.6 ± 2.0	27.6 ± 1.9	27.0 ± 2.0	< 0.001
MCI, n (%)	527 (24.9)	438 (23.4)	89 (36.0)	< 0.001
Dementia onset, n (%)	151 (7.1)	116 (6.2)	35 (14.2)	< 0.001

IADL, instrumental activities of daily living; NCGG-ADL, National Center for Geriatrics and Gerontology Activities of Daily Living; MMSE, Mini-Mental State Examination; MCI, mild cognitive impairment.

Data are expressed as means ± standard deviations or numbers (%)

^*^ P-values are based on the Student’s t-test for continuous variables and the chi-squared test for categorical variables

Table 2 shows the differences in characteristics between participants with and without dementia onset. At baseline, participants who developed dementia were significantly older (*P* < 0.001) and had lower education levels (*P* = 0.004), a higher prevalence of diabetes mellitus (*P* = 0.022), lower global cognitive functions (*P* < 0.001), and a higher prevalence of MCI (*P* < 0.001) than did participants who did not develop dementia.

**Table 2:** Participant characteristics according to dementia onset during the 5-year follow-up

Variables	Dementia onset during the 5-year follow-up		*P*-value ^*^
	Participants without dementia onset *n* = 1,967	Participants with dementia onset *n* = 151	
Age, years	73.0 ± 5.7	79.4 ± 5.0	< 0.001
Sex, men (%)	787 (40.0)	54 (35.8)	0.304
Education, years	11.1 ± 2.3	10.5 ± 2.3	0.004
Hypertension, n (%)	987 (50.2)	80 (53.0)	0.507
Diabetes mellitus, n (%)	271 (13.8)	31 (20.5)	0.022
Heart disease, n (%)	327 (16.6)	21 (13.9)	0.385
MMSE, points	27.6 ± 2.0	26.9 ± 1.9	< 0.001
MCI, n (%)	462 (23.5)	65 (43.0)	< 0.001

MMSE, Mini-Mental State Examination; MCI, mild cognitive impairment.

Data are expressed as means ± standard deviations or numbers (%)

^*^ P-values are based on the Student’s t-test for continuous variables and the chi-squared test for categorical variables

### Predictive validity of IADL ability assessed using the NCGG-ADL for identifying dementia risk

**Fig. 1:**
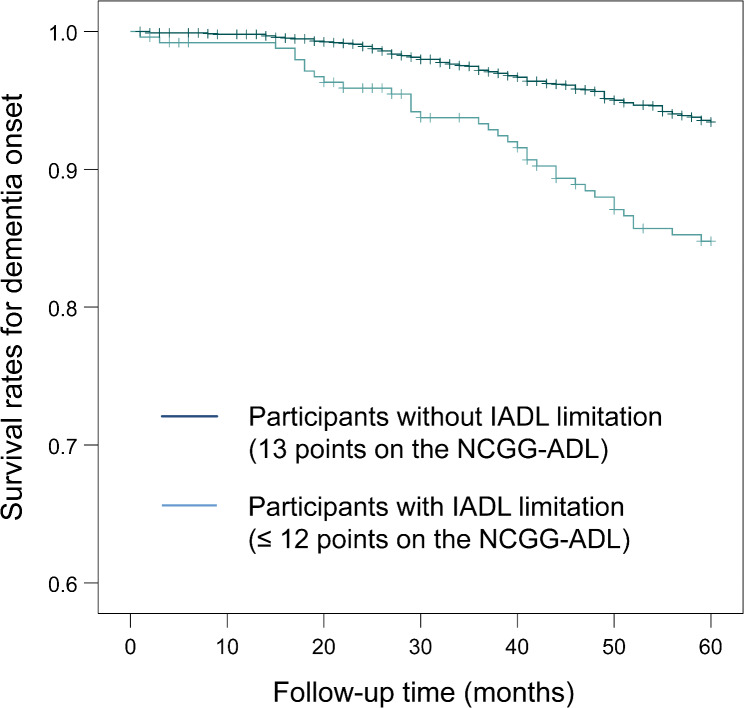
Cumulative survival rates on dementia onset according to IADL. IADL, instrumental activities of daily living; NCGG-ADL, National Center for Geriatrics and Gerontology Activities of Daily Living

Figure 1 shows the Kaplan–Meier survival curve for dementia onset according to IADL ability assessed using the NCGG-ADL. In the log-rank test, participants with IADL limitations (NCGG-ADL ≤ 12 points) showed a significantly higher risk of dementia onset (*P* < 0.001) than did those without IADL limitations (NCGG-ADL 13 points). In the Cox proportional hazards regression analysis, the HRs (95%CIs) of NCGG-ADL score were 0.72 (0.61–0.84) in the crude model, 0.87 (0.71–1.05) in adjusted model 1, and 0.88 (0.73–1.07) in adjusted model 2. In the Cox regression model using the categorized IADL limitation (reference: participants without IADL limitations), the HRs (95% CIs) in those with IADL limitations were 2.47 (1.69–3.60) in the crude model, 1.59 (1.07–2.37) in adjusted model 1, and 1.55 (1.04–2.31) in adjusted model 2 (Table 3). The sensitivity analysis in participants with MMSE ≥ 28 points showed results consistent with those of the main analysis (Supplementary Table 1).

**Table 3:** Hazard ratios and 95% confidence intervals for dementia onset over 5 years

Explanatory variables		Crude model		Adjusted model 1^*^		Adjusted model 2^†^
		HR (95% CI)		HR (95% CI)		HR (95% CI)
*IADL ability based on the NCGG-ADL*						
NCGG-ADL score, points		0.72 (0.61–0.84)		0.87 (0.71–1.05)		0.88 (0.73–1.07)
*IADL limitation based on the NCGG-ADL*						
Participants without IADL limitation		Reference		Reference		Reference
Participants with IADL limitation		2.47 (1.69–3.60)		1.59 (1.07–2.37)		1.55 (1.04–2.31)
*Combination of MCI and IADL limitation*						
Unimpaired cognition without IADL limitation		Reference		Reference		Reference
Unimpaired cognition with IADL limitation		2.49 (1.48–4.18)		1.56 (0.92–2.67)		1.53 (0.90–2.61)
MCI without IADL limitation		2.47 (1.70–3.57)		1.96 (1.35–2.85)		1.87 (1.28–2.74)
MCI with IADL limitation		4.92 (2.89–8.37)		3.03 (1.74–5.27)		2.88 (1.65–5.03)
P-value for trend		< 0.001		< 0.001		< 0.001

HR, hazard ratio; CI, confidence interval; IADL, instrumental activities of daily living; NCGG-ADL, National Center for Geriatrics and Gerontology Activities of Daily Living; MCI, mild cognitive impairment

^*^ Adjusted model 1 was adjusted for age, sex, education, hypertension, diabetes mellitus, and heart disease

^†^ Adjusted model 2 was additionally adjusted for score of Mini-Mental State Examination as global cognitive function at baseline

### Combined impact of MCI and IADL limitations on dementia onset

At baseline, the group classification according to MCI and IADL limitations were as follows: (1) unimpaired cognition without IADL limitations, *n* = 1,433 (67.7%); (2) unimpaired cognition with IADL limitations, *n* = 158 (7.5%); (3) MCI without IADL limitations, *n* = 438 (20.7%); and (4) MCI with IADL limitations, *n* = 89 (4.2%).

**Fig. 2:**
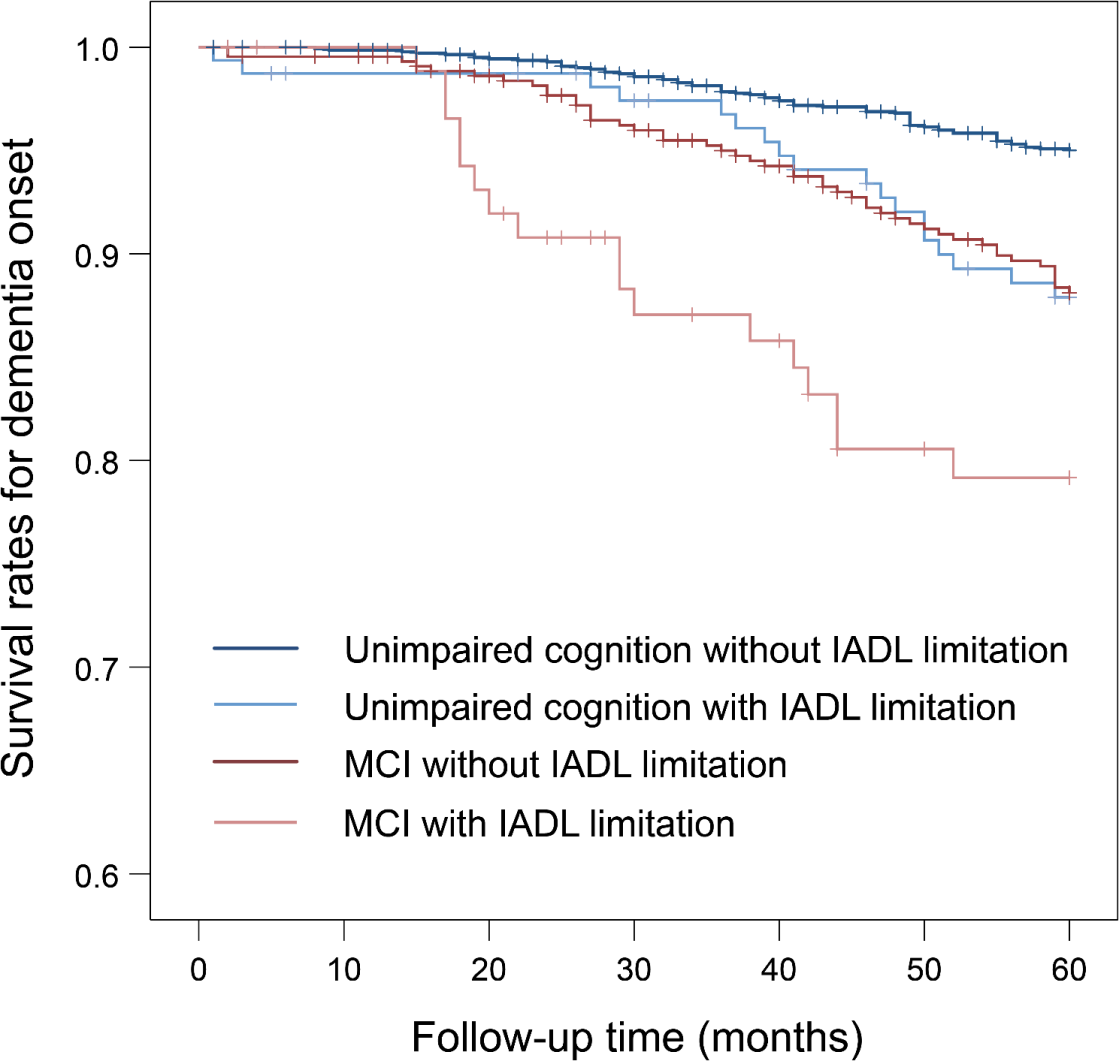
Cumulative survival rates on dementia onset according to MCI and IADL. IADL, instrumental activities of daily living; MCI, mild cognitive impairment

Figure 2 shows the Kaplan–Meier survival curve for dementia onset according to MCI and IADL limitations. In the log-rank test, compared to unimpaired cognition without IADL limitations, significantly higher risks of dementia were observed for unimpaired cognition with IADL limitations (*P* = 0.001), MCI without IADL limitations (*P* < 0.001), and MCI with IADL limitations (*P* < 0.001). Furthermore, the risk of dementia in MCI with IADL limitations was significantly higher than that in MCI without IADL limitations (*P* = 0.041). In the Cox proportional hazards regression analysis (reference: unimpaired cognition without IADL limitations), the HRs (95% CIs) of unimpaired cognition with IADL limitations, MCI without IADL limitations, and MCI with IADL limitations were 1.53 (0.90–2.61), 1.87 (1.28–2.74), and 2.88 (1.65–5.03), respectively, in the full-adjusted model (adjusted model 2), and a significant trend was observed in HRs between the four groups (*P* < 0.001, Table 3).

## Discussion

This population-based longitudinal cohort study confirmed that IADL assessment based on the NCGG-ADL is useful for predicting dementia risk among community-dwelling older adults. Additionally, the coexistence of MCI and IADL limitations was associated with a higher risk of dementia than either condition alone. Older individuals with a combination of MCI and IADL limitations should be recognized as a population at a particularly high risk for dementia.

Several population-based cohort studies have shown that IADL ability predicts dementia onset [3, 8, 28] using validated traditional IADL assessment scales such as the Lawton IADL scale [7]. Our results using the NCGG-ADL support the above evidence and reinforce the importance of IADL assessment in community-dwelling older adults without dementia. Although we did not directly compare the predictive ability of the NCGG-ADL and other IADL scales in this study, the NCGG-ADL, which was adapted to recent lifestyles, including electrical appliances, could be a useful tool for predicting dementia risk. Regarding the dose-response relationship between IADL ability and dementia risk, several previous studies reported that continuous IADL score were associated with dementia risk [3, 28]. On the other hand, NCGG-ADL score as a continuous value was not associated with dementia onset in our fully adjusted regression models, which may suggest that IADL decline may increase dementia risk with a certain threshold. Notably, 88.3% of the study participants showed a full score (13 points) on the NCGG-ADL, indicating that the ceiling effect of the scale may have reduced the statistical power of our analysis. The potential reasons why IADL ability predicts dementia onset are not yet clear; however, several explanations are possible. IADLs are higher-order activities than BADLs and require a more complex neuropsychological organization [4]. Therefore, IADL performance may reflect cognitive performance in daily life and may be sensitive to cognitive decline during the pre-clinical phase. Furthermore, the process of performing IADLs may contain cognitive components that are not captured by neuropsychological tests [3]. IADL ability, which can be assessed with relatively little time and human resources, may provide unique and useful information for detecting dementia risk in community-dwelling older adults without dementia. Additionally, there may be shared pathological conditions that affect both IADL limitation and dementia development. For example, neuropsychiatric disorders are known to increase the risk of both functional limitations [29] and dementia [30] in older adults. However, this study did not collect specific information on neuropsychiatric disorders other than stroke and depression, which were used as exclusion criteria, and additional studies including a more detailed assessment of background diseases or pathologies are required in the future.

Regarding the combination of MCI and IADL limitations, the comorbid group showed a higher risk of dementia onset than either condition alone in this study, which is consistent with previous studies [3, 9]. Fieo et al. examined the temporal relationship between cognitive impairment and IADL limitations and demonstrated that poor cognitive function was associated with more rapid subsequent decline in advanced ADLs. Conversely, limitations in advanced ADLs were associated with more rapid subsequent cognitive decline [31]. Our other study showed that IADL limitations in cognitively normal older adults predicted the future incidence of MCI [32]. Therefore, IADLs may be a consequence of cognitive decline and beneficial for maintaining cognitive function by providing cognitive stimulation. IADL limitations and MCI are common comorbid conditions in older individuals and may have reciprocal relationships [5]. To prevent a negative cycle of IADL limitations and MCI, older individuals with both conditions should receive special attention. However, in this study, there was no significant difference in the dementia risk between those with unimpaired cognition without IADL limitations and those with unimpaired cognition with IADL limitations in the fully adjusted Cox regression model. This result suggests that the impact of IADLs on dementia risk may vary according to the baseline cognitive function level, and further investigation, including a more detailed stratification of the association between IADLs and dementia onset, is warranted.

### Strengths and limitations

The major strength of this study lies in the analysis of well-characterized cohort data, including monthly follow-up for dementia. Additionally, this study incorporated population-based data from older adults without dementia and/or severe cognitive impairment at baseline. Therefore, our findings can be generalized to community-dwelling individuals in primary care settings.

However, this study had several limitations. First, our definition of dementia onset was based on records from Public Health Insurance (the Japanese National Health Insurance and Late-Stage Medical Care System) and Long-Term Care Insurance, and we could not follow the health status of some participants who did not have the above insurance (i.e. those who are covered by the Employees’ Health Insurance). There were some differences in the characteristics of participants who were followed-up by public insurance records (*n* = 2,118) and those without available insurance data (*n* = 496) (shown in Supplementary Table 2); this selection bias may have affected our results. Second, there may have been participants who developed dementia but did not consult a medical or long-term care facility, and this may have led to an underestimation of dementia cases in this study. Furthermore, a certain time lag may exist from actual cognitive decline to the diagnosis of dementia, and the possibility of reverse causation between IADL limitation and dementia onset cannot be completely ruled out. Third, previous studies have demonstrated that the IADL status is influenced by socioeconomic status and residential areas [33]. Further research is required to examine the cross-cultural validity of the NCGG-ADL in diverse populations. Fourth, although we excluded participants with a history of stroke and/or depression to avoid disease-specific effects on dementia and reduce bias in self-reported IADL status, the effects of confounding due to other neuropsychiatric disorders have not been examined in this study.

## Conclusions

This study validated the NCGG-ADL as a screening tool for identifying the dementia risk in community-dwelling older adults. Furthermore, the results suggest that the coexistence of MCI and IADL limitations was associated with a higher dementia onset than either condition alone, suggesting that comorbidities should be monitored carefully to prevent dementia.

## Electronic supplementary material

Below is the link to the electronic supplementary material.

Supplementary Material 1

## Data Availability

The datasets generated and/or analyzed during the current study are not publicly available due to privacy/ethical restrictions but are available from the corresponding author on reasonable request.

## Abbreviations

ADL: Activities of Daily Living
BADL: Basic Activities of Daily Living
CI: Confidence Interval
HR: Hazard Ratio
IADL: Instrumental Activities of Daily Living
ICD–10: International Classification of Disease–10
MCI: Mild Cognitive Impairment
MMSE: Mini–Mental State Examination
NCGG: National Center for Geriatrics and Gerontology
NCGG-ADL: National Center for Geriatrics and Gerontology–Activities of Daily Living
NCGG–FAT: National Center for Geriatrics and Gerontology–Functional Assessment Tool
NCGG–SGS: National Center for Geriatrics and Gerontology–Study of Geriatric Syndromes

## References

[1] World Health Organization. Risk reduction of cognitive decline and dementia: WHO guidelines. World Health Organization; 2019. https://iris.who.int/bitstream/handle/10665/312180/9789241550543-eng.pdf?sequence=17. 31219687

[2] Petersen RC. Clinical practice. Mild cognitive impairment. N Engl J Med. 2011;364(23):2227–34. 10.1056/NEJMcp0910237. 21651394 10.1056/NEJMcp0910237

[3] Di Carlo A, Baldereschi M, Lamassa M, Bovis F, Inzitari M, Solfrizzi V, et al. Daily function as predictor of dementia in cognitive impairment, no dementia (CIND) and mild cognitive impairment (MCI): an 8-year follow-up in the ILSA study. J Alzheimers Dis. 2016;53(2):505–15. 10.3233/jad-160087. 27163817 10.3233/JAD-160087

[4] Pérès K, Chrysostome V, Fabrigoule C, Orgogozo JM, Dartigues JF, Barberger-Gateau P. Restriction in complex activities of daily living in MCI: impact on outcome. Neurology. 2006;67(3):461–6. 10.1212/01.wnl.0000228228.70065.f1. 16894108 10.1212/01.wnl.0000228228.70065.f1

[5] Jekel K, Damian M, Wattmo C, Hausner L, Bullock R, Connelly PJ, et al. Mild cognitive impairment and deficits in instrumental activities of daily living: a systematic review. Alzheimers Res Ther. 2015;7(1):17. 10.1186/s13195-015-0099-0. 25815063 10.1186/s13195-015-0099-0 PMC4374414

[6] Petersen RC, Caracciolo B, Brayne C, Gauthier S, Jelic V, Fratiglioni L. Mild cognitive impairment: a concept in evolution. J Intern Med. 2014;275(3):214–28. 10.1111/joim.12190. 24605806 10.1111/joim.12190 PMC3967548

[7] Lawton MP, Brody EM. Assessment of older people: self-maintaining and instrumental activities of daily living. Gerontologist. 1969;9(3):179–86. 5349366

[8] Pérès K, Helmer C, Amieva H, Orgogozo JM, Rouch I, Dartigues JF, et al. Natural history of decline in instrumental activities of daily living performance over the 10 years preceding the clinical diagnosis of dementia: a prospective population-based study. J Am Geriatr Soc. 2008;56(1):37–44. 10.1111/j.1532-5415.2007.01499.x. 18028344 10.1111/j.1532-5415.2007.01499.x

[9] Luck T, Luppa M, Angermeyer MC, Villringer A, König HH, Riedel-Heller SG. Impact of impairment in instrumental activities of daily living and mild cognitive impairment on time to incident dementia: results of the Leipzig longitudinal study of the aged. Psychol Med. 2011;41(5):1087–97. 10.1017/s003329171000142x. 20667169 10.1017/S003329171000142X

[10] Sikkes SA, Visser PJ, Knol DL, de Lange-de Klerk ES, Tsolaki M, Frisoni GB, et al. Do instrumental activities of daily living predict dementia at 1- and 2-year follow-up? Findings from the development of screening guidelines and diagnostic criteria for predementia Alzheimer’s disease study. J Am Geriatr Soc. 2011;59(12):2273–81. 10.1111/j.1532-5415.2011.03732.x. 22188074 10.1111/j.1532-5415.2011.03732.x

[11] Koyano W, Shibata H, Nakazato K, Haga H, Suyama Y. Measurement of competence: reliability and validity of the TMIG index of competence. Arch Gerontol Geriatr. 1991;13(2):103–16. 10.1016/0167–4943(91)90053-s. 15374421 10.1016/0167-4943(91)90053-s

[12] Nouri F, Lincoln N. An extended activities of daily living scale for stroke patients. Clin Rehabil. 1987;1(4):301. 10.1177/026921558700100409.

[13] Sikkes SA, de Lange-de Klerk ES, Pijnenburg YA, Gillissen F, Romkes R, Knol DL, et al. A new informant-based questionnaire for instrumental activities of daily living in dementia. Alzheimers Dement. 2012;8(6):536–43. 10.1016/j.jalz.2011.08.006. 23102123 10.1016/j.jalz.2011.08.006

[14] Makino K, Lee S, Bae S, Shinkai Y, Chiba I, Shimada H. Predictive validity of a new instrumental activities of daily living scale for detecting the incidence of functional disability among community-dwelling older Japanese adults: A prospective cohort study. Int J Environ Res Public Health. 2020;17(7). 10.3390/ijerph17072291. 10.3390/ijerph17072291 PMC7177260 32235309

[15] Folstein MF, Robins LN, Helzer JE. The Mini-Mental state examination. Arch Gen Psychiatry. 1983;40(7):812. 10.1001/archpsyc.1983.01790060110016. 6860082 10.1001/archpsyc.1983.01790060110016

[16] Shimada H, Makizako H, Tsutsumimoto K, Doi T, Lee S, Suzuki T. Cognitive frailty and incidence of dementia in older persons. J Prev Alzheimers Dis. 2018;5(1):42–8. 10.14283/jpad.2017.29.29405232 10.14283/jpad.2017.29

[17] Makizako H, Shimada H, Park H, Doi T, Yoshida D, Uemura K, et al. Evaluation of multidimensional neurocognitive function using a tablet personal computer: test-retest reliability and validity in community-dwelling older adults. Geriatr Gerontol Int. 2013;13(4):860–6. 10.1111/ggi.12014. 23230988 10.1111/ggi.12014

[18] Shimada H, Tsutsumimoto K, Lee S, Doi T, Makizako H, Lee S, et al. Driving continuity in cognitively impaired older drivers. Geriatr Gerontol Int. 2016;16(4):508–14. 10.1111/ggi.12504. 25953032 10.1111/ggi.12504

[19] Makino K, Raina P, Griffith LE, Lee S, Harada K, Katayama O, et al. Lifetime physical activity and late-life mild cognitive impairment in community-dwelling older adults. J Am Med Dir Assoc. 2024;25(3):488–93. 10.1016/j.jamda.2023.12.006. 38246592 10.1016/j.jamda.2023.12.006

[20] Winblad B, Palmer K, Kivipelto M, Jelic V, Fratiglioni L, Wahlund LO, et al. Mild cognitive impairment–beyond controversies, towards a consensus: report of the international working group on mild cognitive impairment. J Intern Med. 2004;256(3):240–6. 10.1111/j.1365-2796.2004.01380.x. 15324367 10.1111/j.1365-2796.2004.01380.x

[21] Ministry of Health, Labour and Welfare. Health Insurance.: Ministry of Health, Labour and Welfare; [https://www.mhlw.go.jp/english/policy/health-medical/health-insurance/index.html

[22] Shimada H, Doi T, Tsutsumimoto K, Makino K, Harada K, Tomida K, et al. Elevated risk of dementia diagnosis in older adults with low frequencies and durations of social conversation. J Alzheimers Dis. 2024. 10.3233/jad-231420. 38461507 10.3233/JAD-231420

[23] Ikegami N. Public long-term care insurance in Japan. JAMA. 1997;278(16):1310–4. 9343449

[24] Moriyama Y, Tamiya N, Kamimura A, Sandoval F, Luptak M. Doctors’ opinion papers in long-term care need certification in Japan: comparison between clinic and advanced treatment hospital settings. Public Policy Adm Res. 2014;4:31–7.

[25] Ihira H, Sawada N, Inoue M, Yasuda N, Yamagishi K, Charvat H, et al. Association between physical activity and risk of disabling dementia in Japan. JAMA Netw Open. 2022;5(3):e224590. 10.1001/jamanetworkopen.2022.4590. 35348711 10.1001/jamanetworkopen.2022.4590 PMC8965633

[26] Yamamoto T, Kondo K, Hirai H, Nakade M, Aida J, Hirata Y. Association between self-reported dental health status and onset of dementia: a 4-year prospective cohort study of older Japanese adults from the Aichi gerontological evaluation study (AGES) project. Psychosom Med. 2012;74(3):241–8. 10.1097/PSY.0b013e318246dffb. 22408130 10.1097/PSY.0b013e318246dffb

[27] Nishita Y, Makizako H, Jeong S, Otsuka R, Kim H, Obuchi S, et al. Temporal trends in cognitive function among community-dwelling older adults in Japan: findings from the ILSA-J integrated cohort study. Arch Gerontol Geriatr. 2022;102:104718. 10.1016/j.archger.2022.104718. 35605287 10.1016/j.archger.2022.104718

[28] Reppermund S, Brodaty H, Crawford JD, Kochan NA, Draper B, Slavin MJ, et al. Impairment in instrumental activities of daily living with high cognitive demand is an early marker of mild cognitive impairment: the Sydney memory and ageing study. Psychol Med. 2013;43(11):2437–45. 10.1017/s003329171200308x. 23308393 10.1017/S003329171200308X

[29] Reis Júnior WM, Ferreira LN, Molina-Bastos CG, Bispo Júnior JP, Reis HFT, Goulart BNG. Prevalence of functional dependence and chronic diseases in the community-dwelling Brazilian older adults: an analysis by dependence severity and Multimorbidity pattern. BMC Public Health. 2024;24(1):140. 10.1186/s12889-023-17564-w. 38200484 10.1186/s12889-023-17564-w PMC10777626

[30] Ismail Z, Ghahremani M, Amlish Munir M, Fischer CE, Smith EE, Creese B. A longitudinal study of late-life psychosis and incident dementia and the potential effects of race and cognition. Nat Mental Health. 2023;1(4):273–83. 10.1038/s44220-023-00043-x.

[31] Fieo R, Zahodne L, Tang MX, Manly JJ, Cohen R, Stern Y. The historical progression from ADL scrutiny to IADL to advanced ADL: assessing functional status in the earliest stages of dementia. J Gerontol Biol Sci Med Sci. 2018;73(12):1695–700. 10.1093/gerona/glx235. 10.1093/gerona/glx235 PMC6230209 29244089

[32] Makino K, Lee S, Bae S, Shinkai Y, Chiba I, Shimada H. Relationship between instrumental activities of daily living performance and incidence of mild cognitive impairment among older adults: A 48-month follow-up study. Arch Gerontol Geriatr. 2020;88:104034. 10.1016/j.archger.2020.104034. 32109693 10.1016/j.archger.2020.104034

[33] Zhang X, Dupre ME, Qiu L, Zhou W, Zhao Y, Gu D. Urban-rural differences in the association between access to healthcare and health outcomes among older adults in China. BMC Geriatr. 2017;17(1):151. 10.1186/s12877-017-0538-9. 28724355 10.1186/s12877-017-0538-9 PMC5516359

