# Supplementary material for: Prediction of dementia risk by instrumental activities of daily living limitations and its impact on dementia onset in combination with mild cognitive impairment: a population-based longitudinal study
Source: BMC Public Health. 2025 Apr 25;25:1535. doi: 10.1186/s12889-025-22788-z (PMC12023375; doi:10.1186/s12889-025-22788-z)
Supplement: Supplementary file 1 — Supplementary Material 1 [file 12889_2025_22788_MOESM1_ESM.docx]

**Supplementary Table 1. Sensitivity analysis on IADL and dementia onset in participants with MMSE ≥28 points**

| **Explanatory variables** |  | **Crude model** |  | **Adjusted model 1^*^** |  | **Adjusted model 2^†^** |
| --- | --- | --- | --- | --- | --- | --- |
|  |  | **HR (95% CI)** |  | **HR (95% CI)** |  | **HR (95% CI)** |
| *IADL ability based on the NCGG-ADL* | | | | | | |
| NCGG-ADL score, points |  | 0.66 (0.48–0.90) |  | 0.82 (0.56–1.21) |  | 0.83 (0.56–1.23) |
| *IADL limitation based on the NCGG-ADL* | | | | | | |
| Participants without IADL limitation |  | Reference |  | Reference |  | Reference |
| Participants with IADL limitation |  | 2.81 (1.55–5.08) |  | 1.80 (0.94–3.42) |  | 1.75 (0.92–3.34) |
| HR, hazard ratio; CI, confidence interval; IADL, instrumental activities of daily living; NCGG-ADL, National Center for Geriatrics and Gerontology Activities of Daily Living.  ^*^ Adjusted model 1 was adjusted for age, sex, education, hypertension, diabetes mellitus, and heart disease.  ^†^ Adjusted model 2 was additionally adjusted for score of Mini-Mental State Examination as global cognitive function at baseline. | | | | | | |

**Supplementary Table 2. Comparison of baseline characteristics between participants followed by public insurance records and those without available insurance data**

| **Variables** | **Participants with**  **public insurance records**  ***n* = 2,118** | **Participants without**  **available insurance data**  ***n* = 496** | **P-value** ^*^ |
| --- | --- | --- | --- |
| Age, years | 73.5 ± 5.9 | 69.2 ± 3.3 | <0.001 |
| Sex, men (%) | 841 (39.7) | 272 (54.8) | <0.001 |
| Education, years | 11.0 ± 2.3 | 11.7 ± 2.4 | <0.001 |
| Hypertension, n (%) | 1,067 (50.4) | 216 (43.5) | 0.006 |
| Diabetes mellitus, n (%) | 302 (14.3) | 72 (14.5) | 0.883 |
| Heart disease, n (%) | 348 (16.4) | 62 (12.5) | 0.030 |
| MMSE, points | 27.6 ± 2.0 | 27.9 ± 1.9 | 0.001 |
| MCI, n (%) | 527 (24.9) | 111 (22.4) | 0.243 |
| IADL limitation, n (%) | 247 (11.7) | 66 (13.3) | 0.310 |
| MMSE, Mini-Mental State Examination; IADL, instrumental activities of daily living; MCI, mild cognitive impairment.  Data are expressed as means ± standard deviations or numbers (%).  ^*^ P-values are based on the Student’s t-test for continuous variables and the chi-squared test for categorical variables. | | | |
